# Higher level of neutrophil-to-lymphocyte is associated with severe COVID-19

**DOI:** 10.1017/S0950268820001557

**Published:** 2020-07-09

**Authors:** Man Kong, Hongmei Zhang, Xiaocui Cao, Xiaoli Mao, Zhongxin Lu

**Affiliations:** 1 Department of Medical Laboratory, The Central Hospital of Wuhan, Tongji Medical College, Huazhong University of Science and Technology, Wuhan, Hubei 430014, China; 2 School of Laboratory Medicine, Hubei University of Chinese Medicine, Wuhan 430065, China; 3 Cancer Research Institute of Wuhan, The Central Hospital of Wuhan, Tongji Medical College, Huazhong University of Science and Technology, Wuhan 430014, China; 4 Key Laboratory for Molecular Diagnosis of Hubei Province, The Central Hospital of Wuhan, Tongji Medical College, Huazhong University of Science and Technology, Wuhan 430014, China

**Keywords:** COVID-19, immune response, NLR, severe illness

## Abstract

In December 2019, cases of severe coronavirus 2019 (COVID-19) infection rapidly progressed to acute respiratory failure. This study aims to assess the association between the neutrophil-to-lymphocyte ratio (NLR) and the incidence of severe COVID-19 infection. A retrospective cohort study was conducted on 210 patients with COVID-19 infection who were admitted to the Central Hospital of Wuhan from 27 January 2020 to 9 March 2020. Peripheral blood samples were collected and examined for lymphocyte subsets by flow cytometry. Associations between tertiles of NLR and the incidence of severe illness were analysed by logistic regression.

Of the 210 patients with COVID-19, 87 were diagnosed as severe cases. The mean NLR of the severe group was higher than that of the mild group (6.6 *vs.* 3.3, *P* < 0.001). The highest tertile of NLR (5.1–19.7) exhibited a 5.9-fold (95% CI 1.3–28.5) increased incidence of severity relative to that of the lowest tertile (0.6–2.5) after adjustments for age, diabetes, hypertension and other confounders. The number of T cells significantly decreased in the severe group (0.5 *vs.* 0.9, *P* < 0.001). COVID-19 might mainly act on lymphocytes, particularly T lymphocytes. NLR was identified as an early risk factor for severe COVID-19 illness. Patients with increased NLR should be admitted to an isolation ward with respiratory monitoring and supportive care.

## Introduction

In December 2019, an ongoing outbreak of unexplained pneumonia in Wuhan drew attention globally [1]. Deep gene sequencing confirmed that the patients with pneumonia were infected with a novel *β*-coronavirus, which was identified as a severe acute respiratory syndrome coronavirus 2 (SARS-CoV-2) by the International Committee on Taxonomy of Viruses. The pneumonia caused by SARS-CoV-2 was referred to as coronavirus disease 2019 (COVID-19) by the World Health Organization on 30 January 2020 [2]. As of 9 March 2020, 80 735 confirmed cases, including 5111 severe cases and 3119 deaths, were reported in China.

Most patients with COVID-19 infection reportedly experienced mild cold symptoms, including fever, cough and fatigue. Although computed tomography (CT) of the chest indicated increased infection after 3–5 days, the prognosis was good [3]. In patients with severe infection, the disease developed rapidly into acute respiratory distress syndrome, coagulopathy, septic shock and even death [4, 5]. For mild cases, symptomatic treatment and general isolation were provided at centralised isolation points or non-critical designated hospitals. However, for severe cases, efforts were directed towards preventing the development of critical diseases; risk factors were identified as early as possible; appropriate supportive care was provided; and transfer to intensive special hospital was coordinated if necessary to reduce mortality. Rational use of medical resources was also implemented.

A study indicated that large amounts of pro-inflammatory cytokines in serum were associated with pulmonary inflammation and extensive lung damage in COVID-19, similar to severe acute respiratory syndrome (SARS) and Middle East Respiratory Syndrome coronavirus (MERS-COV) infection [6]. The current study aimed to investigate the association between different laboratory data (including lymphocyte subsets and inflammatory biomarkers) and clinical characteristics of hospitalised patients with mild and severe COVID-19 infection to reveal a potentially useful prognostic factor associated with severe morbidity.

## Methods

### Study design and participants

This study was a retrospective single-centre study among patients treated at the Central Hospital of Wuhan, a specific hospital for the treatment of patients with COVID-19. Lymphocyte subset analysis was conducted on the patients who were included in the final analysis and admitted from 27 January 2020 to 9 March 2020. The clinical diagnosis of viral pneumonia was initially based on their clinical symptoms, including fever, cough or respiratory illness and typical changes in chest CT. All patients in this study lived in Wuhan during the outbreak period of COVID-19. This study was approved by the Ethics Review Committee of the Central Hospital of Wuhan. All patients regularly signed informed consent when they were admitted to Wuhan Union Hospital, China.

The clinical classification of COVID-19 was in accordance with the Diagnosis and Treatment Protocol for COVID-19 (Trial Version 7) issued by the National Health Committee of the People's Republic of China (http://www.nhc.gov.cn/). The condition was considered as severe-type COVID-19 when one of the following criteria was present: (1) Respiratory distress with respiratory rate >30/min; (2) oxygen saturation ≤93% in the resting state; or (3) arterial blood oxygen partial pressure (PaO_2_)/oxygen concentration (FiO_2_)≤300 mmHg (1 mmHg = 0.133 kPa). If follow-up of these cases revealed progression into cases requiring admission to intensive care unit (ICU), the prognosis was considered very poor.

### Data collection

Throat swab samples were collected from all patients for COVID-19 viral nucleic acid detection via real-time reverse transcription–polymerase chain reaction assay. This test was performed in a clinical laboratory at the Central Hospital of Wuhan in accordance with the Chinese Center for Disease Control and Prevention protocol.

The data collected included demographic data, symptoms, chronic disease, medical history, outcome events from the Electronic Medical Record system and findings from the laboratory information system. Laboratory results included blood routine, coagulation routine, biochemical indicators, infection-related biomarkers and lymphocyte subsets. The percentages of the lymphocyte subsets were analysed using the BD FACSCanto flow cytometer. The laboratory data of some patients were missing because of delayed results or the absence of test types. The data that support the findings of this study are available from the Central Hospital of Wuhan. Restrictions apply to the availability of these data, which were used under licence for this study.

### Statistical analysis

Descriptive statistics were presented as means ± s.d. for continuous variables and as percentages for categorical variables. Student's *t*-test was used to compare normally distributed continuous variables, whereas the Mann–Whitney *U*-test was applied for data not obeying a normal distribution, which was represented by the median (lower quartile, upper quartile). The non-parametric Chi-squared test was used to compare categorical variables. A linear regression model was used to analyse the correlation between neutrophil-to-lymphocyte ratio (NLR) levels and the incidence of severe illness. Multivariate logistic regression was used in the three models to obtain the odds ratios (ORs) and the corresponding 95% confidence intervals (95% CIs) between the tertiles of NLR and the incidence of severe cases. ORs were not adjusted for any variable in the crude model. Model 1 was adjusted for age, C-reactive protein, interleukin-6, procalcitonin, diabetes and hypertension. Subsequently, lactic dehydrogenase (LDH), white blood count (WBC), D-dimer, CD4+ T cells and CD8+ T cells were further adjusted. The lowest tertile of NLR was used as the reference under each model to calculate the ORs and the corresponding 95% CIs. All data were analysed using SPSS version 21 (SPSS Inc., Chicago, IL). *P* < 0.05 was regarded as statistically significant in all statistical analyses.

## Results

### Demographics and clinical characteristics

Table 1 lists the demographic and clinical characteristics of COVID-19 infection. Among the 210 patients diagnosed with COVID-19, 87 (41.4%) were categorised into the severe group and 123 (58.6%) into the mild group upon admission. In the severe group, 38 (43.7%) progressed into ICU or even death; in the mild group, only 1 (0.8%) developed into a critical case. Compared with patients in the mild group, patients in the severe group were older (67.9 ± 12.3 *vs.* 53.2 ± 15.6, *P* = 0.005), particularly those older than 70 years (39.1%). No significant difference in the proportion of women was found between the severe group (50.5%) and the mild group. A total of 72 (82.8%) severe cases and 82 (66.7%) mild cases had a fever, and a significant difference in body temperature was determined between the two groups. Compared with mild cases, severe cases were more likely to experience mild shortness of breath (*P* < 0.001), myalgia (*P* = 0.009), fatigue (*P* = 0.011) and chest congestion (*P* < 0.001). Meanwhile, 99 (47.1%) patients had at least one underlying comorbidity, and the proportion of severe cases with underlying comorbidity was higher than that of mild cases (65.5% *vs.* 34.1%, *P* < 0.001). Comparison between the severe cases and the mild cases indicated significant differences in diabetes (*P* = 0.004), hypertension (*P* < 0.001), chronic renal disease (*P* = 0.024) and tumour (*P* = 0.007), but not in cardiovascular disease, hyperlipidaemia, tuberculosis and chronic obstructive pulmonary disorder (COPD).

**Table 1.** Demographic and baseline characteristics of patients with COVID-19 (*N* = 210)

Patient characteristics	All patients *N* = 210	Mild *N* = 123	Severe *N* = 87	*P*
Age (years)	59.3 ± 16.0	53.2 ± 15.6	67.9 ± 12.3	0.005
<30	11 (5.2%）	11 (8.9%）	0 (0.0%）	<0.001
30–49	43 (20.4%）	38 (30.9%）	5 (5.7%）	
50–69	108 (51.4%）	60 (48.8%）	48 (55.2%）	
≥70	58 (27.6%）	14 (11.4%）	34 (39.1%）	
Gender				0.592
Male	104 (49.5%)	59 (48.0%)	45 (51.7%)	
Female	106 (50.5%)	64 (52.0%)	42 (48.3%)	
Signs and symptoms				
Fever (highest temperature, °C)				<0.001
>39	45 (21.4%)	14 (11.4%)	31 (35.6%)	
38–39	62 (29.5%)	37 (30.1%)	25 (28.7%)	
37.3–38	47 (22.4%)	31 (25.2%)	16 (18.4%)	
<37.3	56 (26.7%)	41 (33.3%)	15 (17.2%)	
Sputum production	79 (37.6%)	44 (35.8%)	35 (40.2%)	0.511
Dry cough	52 (24.8%)	33 (26.8%)	19 (21.8%)	0.409
Mild shortness of breath	30 (14.3%)	4 (3.3%)	26 (29.9%)	<0.001
Headache	21 (10.0%)	10 (8.1%)	11 (12.6%)	0.283
Myalgia	32 (15.2%)	12 (9.8%)	20 (23.0%)	0.009
Fatigue	71 (33.8%)	33 (26.8%)	38 (43.7%)	0.011
Nausea and vomiting	9 (4.3%)	4 (3.3%)	5 (5.7%)	0.379
Diarrhoea	19 (9.0%)	9 (7.3%)	10 (11.5%)	0.299
Chest congestion	64 (30.5%)	26 (21.1%)	38 (43.7%)	<0.001
Chronic medical illness				
Comorbidity	99 (47.1%)	42 (34.1%)	57 (65.5%)	<0.001
Diabetes	27 (12.9%)	9 (7.3%)	18 (20.7%)	0.004
Hypertension	79 (37.6%)	32 (26.0%)	47 (54.0%)	<0.001
Cardiovascular disease	20 (9.5%)	9 (7.3%)	11 (12.6%)	0.195
Hyperlipidaemia	5 (2.4%)	2 (1.6%)	3 (3.4%)	0.394
Chronic renal disease	9 (4.3%)	2 (1.6%)	7 (8.0%)	0.024
Tuberculosis	1 (0.5%)	1 (0.8%)	0 (0.0%)	0.399
COPD	2 (1.0%)	1 (0.8%)	1 (1.1%)	0.805
Tumour	5 (2.4%)	0 (0.0%)	5 (5.7%)	0.007
Poor prognosis (yes)	39 (18.6%)	1 (0.8%)	38 (43.6%)	<0.001

COPD, chronic obstructive pulmonary disorder. Data expressed as the mean ± standard deviation, number (%). *P*-values for continuous data were obtained from Student's *t*-test, and *P*-values for categorical data were obtained from the chi-square test. COVID-19, coronavirus disease 2019.

### Laboratory findings

The results of the peripheral blood test of the patients on the day of hospital admission are listed in Table 2. Absolute WBCs and neutrophil counts were significantly higher in the severe group than in the mild group (6.1 *vs.* 5.3, *P* = 0.002 and 4.3 *vs.* 3.1, *P* < 0.001, respectively), whereas the absolute lymphocyte count was significantly lower in the severe group than in the mild group (0.8 *vs.* 1.2, *P* < 0.001). No significant differences in monocyte, haemoglobin and platelet count were found. With regard to the coagulation of all patients, activated partial thrombin time (APTT) and D-dimer were significantly elevated in the severe group (29.9 *vs.* 28.9, *P* = 0.005 and 0.7 *vs.* 0.4, *P* < 0.001, respectively). Compared with the mild group, most patients in the severe group showed higher levels of infection-related indicators, such as procalcitonin (0.07 *vs.* 0.05, *P* < 0.001), C-reactive protein (3.2 *vs.* 0.6, *P* < 0.001), interleukin-6 (9.4 *vs.* 3.2, *P* < 0.001) and erythrocyte sedimentation rate (52.0 *vs.* 34.0, *P* < 0.001), indicating that inflammation was more prominent in severe cases. Other significant abnormal findings related to blood biochemistry included Gamma-glutamyl transferase (GGT), hydroxybutyrate dehydrogenase (HBDH), lactic dehydrogenase (LDH) and blood urea nitrogen (BUN), which could be a direct influence of the COVID-19 virus or an indirect influence of hypoxia. The lymphocyte subsets in all cases were then analysed. The total B cells, NK cells and T cells were significantly lower in the severe group than in the mild group (0.7 *vs.* 1.2 *P* < 0.001); specifically, the CD3 cell count was lower in the severe group than in the mild group (0.4 *vs.* 0.7 *P* < 0.001). No significant differences in B cell count and NK cell count were found between the two groups. The different subsets of T cells were further analysed. Both the suppressor T cells (CD3 + CD8+) (0.2 *vs.* 0.3 *P* < 0.001) and helper T cells (CD3 + CD4+) (0.2 *vs.* 0.5 *P* < 0.001) were significantly lower in the severe group than in the mild group.

**Table 2.** Laboratory findings of patients with COVID-19 (*N* = 210)

Laboratory findings	All patients *N* = 210	Mild *N* = 123	Severe *N* = 87	*P*
White blood cell count, × 10^9^/l	5.6 (3.3–7.9)	5.3 (3.4–7.2)	6.1 (3.3–8.9)	0.002
Neutrophils, × 10^9^/l	3.6 (2.6–5.0)	3.1 (2.4–4.3)	4.3 (2.9–5.9)	<0.001
Lymphocytes, × 10^9^/l	1.0 (0.6–1.4)	1.2 (0.9–1.8)	0.8 (0.5–1.0)	<0.001
Monocyte, × 10^9^/l	0.3 (0.2–0.5)	0.3 (0.2–0.5)	0.3 (0.2–0.5)	0.738
Haemoglobin, g/l	126.2 (107.5–144.9)	128.4 (111.7–145.1)	122.9 (102.1–143.7)	0.200
NLR	4.7 (0.9–9.5)	3.3 (1.0–3.4)	6.6 (2.1–11.1)	<0.001
Platelet count, × 10^9^/l	197.0 (116.6–277.4)	203.2 (123.2–283.2)	188.4 (107.1–269.7)	0.642
PT, s	11.6 (10.3–12.9)	11.5 (10.2–12.8)	11.8 (110.7–12.9)	0.845
INR	1.0 (0.9–1.1)	1.0 (0.9–1.1)	1.0 (0.9–1.1)	1.000
APTT, s	29.3 (23.2–35.4)	28.9 (23.2–34.6)	29.9 (23.4–36.4)	0.005
TT, s	16.5 (15.0–18.0)	16.5 (15.0–18.0)	16.7 (15.1–18.3)	0.227
D-dimer, μg/ml	0.6 (0.3–1.3)	0.4 (0.2–0.8)	0.7 (0.5–1.9)	<0.001
ALT, U/l	20.2 (14.3–31.0)	20.1 (13.8–32.3)	20.1 (14.4–29.7)	0.631
AST, U/l	22.2 (16.8–30.4)	20.6 (16.3–29.6)	23.0 (17.8–32.8)	0.118
GGT, U/l	21.1 (14.0–37.1)	19.0 (12.0–35.1)	26.0 (16.3–26.0)	0.013
TP, g/l	66.3 (58.8–73.8)	67.0 (59.8–74.2)	65.4 (57.5–73.3)	0.811
HBDH, U/l	176.1 (77.1–275.1)	146.4 (96.3–196.5)	217.5 (86.7–348.3)	<0.001
LDH, U/l	223.6 (97.1–350.1)	185.8 (117.6–254.0)	276.2 (111.2–441.2)	<0.001
BUN, μmol/l	4.6 (3.5–6.0)	4.3 (3.4–5.5)	5.1 (4.0–6.6)	<0.001
Creatinine, μmol/l	67.7 (51.5–84.0)	65.1 (51.3–80.0)	70.1 (54.2–88.1)	0.129
C-reactive protein, mg/dl	1.2 (0.1–4.6)	0.6 (0.1–3.3)	3.2 (0.6–6.1)	<0.001
Procalcitonin, ng/ml	0.05 (0.04–0.09)	0.05 (0.04–0.06)	0.07 (0.05–0.19)	<0.001
Interleukin-6, pg/ml	4.3 (1.8–10.3)	3.2 (1.6–6.2)	9.4 (2.9–29.5)	<0.001
Erythocyte sedimentation rate, mm/h	44.0 (14.0–59.0)	34.0 (10.0–48.3)	52.0 (29.5–76.0)	0.006
Lymphocyte subsets				
T cells + B cells + NK cells/l	0.9 (0.6–1.4)	1.2 (0.9–1.7)	0.7 (0.5–0.9)	<0.001
B cells (CD3-CD19+)/l	0.1 (0.1–0.2)	0.1 (0.1–0.2)	0.1 (0.05–0.1)	0.082
NK cells (CD3-CD16 + CD56+)/l	0.1 (0.1–0.2)	0.2 (0.1–0.3)	0.1 (0.1–0.2)	0.074
T cells (CD3 + CD19−)/l	0.7 (0.4–1.0)	0.9 (0.6–1.3)	0.5 (0.3–0.7)	<0.001
Th cells (CD3 + CD4+)/l	0.4 (0.2–0.6)	0.5 (0.3–0.8)	0.2 (0.1–0.4)	<0.001
Ts cells (CD3 + CD8+)/l	0.2 (0.1–0.4)	0.3 (0.2–0.5)	0.2 (0.1–0.3)	<0.001
Th/Ts	1.8 (0.9–2.9)	2.0 (1.0–1.1)	1.5 (0.7–2.3)	0.030

PT, prothrombin time; INR, international normalised ratio; APTT, activated partial thrombin time; TT, thrombin time; NLR, neutrophil-to-lymphocyte ratio; ALT, alanine aminotransferase; AST, aspartate aminotransferase; GGT, Gamma-glutamyl transferase; TP, total protein; HBDH, hydroxybutyrate dehydrogenase; LDH, lactic dehydrogenase; BUN, blood urea nitrogen.

Data expressed as the mean ± standard deviation, median (lower quartile, upper quartile). *P*-values for continuous data conforming to normal distribution were obtained using Student's *t*-test. *P*-values for continuous data not conforming to normal distribution were obtained using the Mann−Whitney *U*-test. COVID-19, coronavirus disease 2019.

### Relationship between NLR and severe illness incidence

Linear regression analysis was used to assess the relationship between NLR and the incidence of severe illness in all participants (Table 3). NLR was positively correlated with the incidence of severe illness (*β* = 0.056, *P* = 0.000).

**Table 3.** Linear regression of NLR on the incidence of severe COVID-19 infection (*N* = 210)

	*β*	*P*-value
NLR	0.056	0.000

NLR, neutrophil-to-lymphocyte ratio; COVID-19, coronavirus disease 2019.

### Independent associations between NLR and the incidence of severe cases

The potential of the aforementioned parameters as potential prognostic predictors of case severity was evaluated. NLR was significantly higher in the severe group than in the mild group (6.6 *vs.* 3.3 *P* < 0.001) (Table 2). Thus, NLR was selected as a potential predictive factor and further analysed. Multivariable logistic regression was used to analyse the correlation between NLR levels and the progress of severe cases (Table 4). NLR was divided into tertiles. The lowest NLR level was used as the reference, and the categorical variables were analysed. The unadjusted logistic regression analysis indicated that upper categorical levels had a statistically significant higher risk of severity than that of lower levels: OR 95% CI 4.1 (1.7–10.2) and OR 95% CI 10.1 (4.0–25.6), respectively. After adjustments for age, C-reactive protein, interleukin-6, procalcitonin, diabetes and hypertension, the highest tertile remained statistically significant, and the relative risk was OR 7.8, 95% CI 2.3–26.4. Further adjustments for other factors, including LDH, WBC, D-dimer, CD4+ T cells and CD8+ T cells showed that the highest level of relative risk (OR 5.9, 95% CI 1.3–28.5) remained significant.

**Table 4.** Association between incidence of severe COVID-19 with NLR by logistic regression (*N* = 210)

NLR	Severe/Total (%)	Crude OR (95% CIs)	Model 1 OR (95% CIs)	Model 2 OR (95% CIs)
Tertile 1 (0.6–2.5)	9 (10.3%)	1 (reference)	1 (reference)	1 (reference)
Tertile 2 (2.6–5.0)	32 (36.8%)	4.1 (1.7–10.2)	2.5 (0.8–7.5)	1.6 (0.5–5.9)
Tertile 3 (5.1–19.7)	46 (52.9%)	10.1 (4.0–25.6)	7.8 (2.3–26.4)	5.9 (1.3–28.5)

Data are presented as *n* (%) or OR (95% CI). Model 1 was adjusted for age, C-reactive protein, interleukin-6, procalcitonin, diabetes and hypertension. Model 2 was further adjusted for LDH, WBC, D-dimer, CD4+ T cells, CD8+ T cells. COVID-19, coronavirus disease 2019; NLR, neutrophil-to-lymphocyte ratio.

## Discussion

This study included 210 patients infected with COVID-19. Their clinical characteristics and laboratory findings were analysed. Compared with the mild group, the severe group was older, mostly had a high fever and had at least one underlying disorder. These clinical features were mostly similar to those in previous studies [1, 7]. This finding suggested that owing to their weakened immune function, older than younger patients with chronic diseases were more likely to be infected with COVID-19.

Early identification of risk factors for severe patients is vital to afford appropriate supportive care or access to ICU if necessary. Severe cases presented lower lymphocyte counts and higher neutrophil levels. The severe group also showed elevated biomarkers for infection. As a widely used factor for systemic infection and inflammation, NLR was used to assess the severity of bacterial infection and the clinical prognosis of pneumonia [8–10]. A study (*n* = 61) recently reported that NLR was the most useful factor affecting the incidence of severe COVID-19 and indicated the incidence of severe illness with NLR ≥ 3.13 [11]. In this study, the patients in the highest NLR tertile presented a 5.9-fold increased risk of incidence of severe COVID-19 after adjustments for potential confounders were applied. Qin *et al*. indicated that NLR increased in several patients with COVID-19 infection, and surveillance of NLR helped in the diagnosis and treatment of COVID-19 infection in the early stages, which was consistent with our findings [12]. An elevated NLR was also found in an early cohort (*n* = 138) with higher neutrophil counts and marked lymphopenia in severe cases [7]. Therefore, NLR could be a useful factor in reflecting the degree of imbalance between inflammatory and immune responses in patients with COVID-19.

The biological mechanism underlying this association has yet to be determined, and several plausible explanations exist. One of the most convincing explanations is based primarily on the physiological link between neutrophilia and lymphopenia with systemic inflammation and stress. Another explanation is that neutrophils are the important cellular components of the host defenses in the innate immune system, whereas lymphocytes are considered as the major cells involved in adaptive immunity [9]. Lymphocytes play a key role in the regulation of inflammatory response, and sustained reduction in severe cases is associated with the non-resolution of inflammation [13]. The mechanism underlying this regulation requires further research.

A well-coordinated innate immune response is known to be the first line of defense against viral infections. However, when the first line of defense is dysregulated, excessive inflammatory cell infiltration, inflammatory storm and even death may occur [14]. Previous studies on SARS-COV and MERS-COV showed that T cells, particularly CD4+ and CD8+ T cells, played a crucial role in inhibiting or weakening overactive innate immune responses during viral infection [14–16]. CD4+ T cells coordinate the deletion and amplification of immune cells to regulate immune responses. CD4+ T cells facilitate virus-specific antibody production via the T-dependent activation of B cells [17]. Moreover, CD8+ T cells primarily exert their effects via two mechanisms – cytokine secretion and cytolytic activities against target cells [18]. Secretion of cytokines such as IFN-*γ* is essential to resist viral and bacterial infections [19]. Zhao *et al*. indicated that CD8+ T cells played a crucial role in viral clearance and immune-mediated injury in most infiltrative inflammatory cells in the pulmonary interstitium [20]. A comparison between B cell-deficient mice and T cell-deficient mice showed that T cells in lungs infected with MERS-COV survived and killed virus-infected cells [21]. These data emphasise the importance of T lymphocytes rather than B cells in controlling the pathogenesis and outcomes of SARS-COV and MERS-COV infection. In the current study, T lymphocytes decreased more in the group with severe COVID-19, similar to SARS-COV and MERS-COV. COVID-19 may attack T cells and destroy our immune system, leading to serious infection. The severity of pathological damage during SARS-COV and MERS-COV was related to the extensive infiltration of pulmonary neutrophils and the increase in neutrophils in peripheral blood [22]. Therefore, COVID-19 could mainly act on lymphocytes, particularly T lymphocytes.

On the basis of the current study, patients with COVID-19 who are suffering from pneumonia and those with increased NLR should be admitted to an isolation ward with respiratory monitoring and supportive care rather putting them into centralised isolation. This finding should largely reduce the progression of critical illness caused by untimely treatment to reduce mortality. Our study has several advantages. One is the previously determined biological plausibility of a strong association between NLR and the risk of incidence of severe cases. The second is the analysis, which eliminates several potential confounding variables to avoid bias. This study also has some limitations: it was a small-sized, single-centre and retrospective study (a larger cohort would be better to eliminate potential bias), and for some patients, repeated measurement data were provided on the first day. The first data point was always used, resulting in potentially incomplete information on variations in intraday cell count.

## Conclusions

COVID-19 could mainly affect lymphocytes, especially T lymphocytes. NLR was an early risk factor affecting the prognosis of patients with severe COVID-19 illness. Patients with a higher NLR should be admitted to an isolation ward with respiratory monitoring and supportive care.
